# Ocular Myasthenia Gravis As Unilateral Ptosis and External Ophthalmoplegia: A Case Report

**DOI:** 10.7759/cureus.56337

**Published:** 2024-03-17

**Authors:** Shafiq Tanveer, Asna Tahir, Obaid Ahmad, Kainat Bibi, Samreen Khan

**Affiliations:** 1 Ophthalmology, Khyber Medical College/Khyber Teaching Hospital, Peshawar, PAK; 2 Ophthalmology, Hayatabad Medical Complex, Peshawar, PAK; 3 Internal Medicine, Ayub Teaching Hospital Complex, Abbottabad, PAK; 4 Pediatrics, Khyber Medical College/Khyber Teaching Hospital, Peshawar, PAK

**Keywords:** unusual presentations, pediatric ophthalmology, myasthenia gravis, case report, autoimmune diseases

## Abstract

Myasthenia gravis (MG) is an autoimmune disorder characterized by fluctuating weakness and fatigue in ocular, bulbar, limb, or respiratory muscles. Initially, more than half of MG patients experience isolated ocular symptoms, such as ptosis, diplopia, or muscle paresis. This case report presents a unique occurrence of MG in a four-year-old female, showcasing a two-year history of sudden onset, persistent yet fluctuating unilateral ptosis accompanied by exo-deviation and adduction deficit in the right eye. No diplopia or systemic features were observed. Positive findings in tests, including the ice pack test, Cogan twitch sign, fatiguability, and neostigmine test, indicated ocular myasthenia. Electromyography revealed a decremental response, while anti-acetylcholine antibodies showed borderline results. Computed tomography of the brain ruled out central causes, and routine laboratory testing yielded normal results. Treatment with pyridostigmine and corticosteroids led to significant improvement in symptoms. This case emphasizes the diverse presentation of MG in ophthalmology, with ocular signs serving as indicators in approximately half of the cases. Early diagnosis and prompt treatment are crucial for enhancing long-term prognosis. Emergency physicians should consider MG as a potential cause for unilateral ocular symptoms after excluding central causes. Accurate diagnosis and comprehensive management of MG are complex yet essential for ensuring optimal patient health.

## Introduction

Myasthenia gravis (MG) is an autoimmune disorder causing muscle fatigue due to acetylcholine receptor deficiency from circulating antibodies. Ocular MG (OMG) involves eye muscles, presenting as ptosis, diplopia, or ophthalmoplegia. The mortality rate without treatment is 25-31%, now reduced to 4% with current therapies [1]. In the pediatric population, the incidence of OMG is 3-9.1 cases per million, and it mostly occurs in children of Asian ethnicity [2]. Treatments include cholinesterase inhibitors, thymectomy, plasmapheresis, corticosteroids, and immunosuppressive drugs. The Tensilon test is the gold standard for confirming the diagnosis of OMG. Approximately 85% of MG patients have anti-acetylcholine receptor (AChR) antibodies, and 30-40% of AChR-negative cases exhibit anti-muscle-specific kinase (anti-MuSK) antibodies, more prevalent in women [3]. Timely diagnosis and treatment improve prognosis. This case describes an unusual presentation of OMG characterized by persistent unilateral ptosis with exo-deviation, responding well to standard therapy for this variant of MG.

## Case presentation

A four-year-old girl with a two-year history of upper eyelid drooping (ptosis) and outward deviation (exotropia) in the right eye presented to a tertiary care hospital in Peshawar. According to her father, she was previously healthy but developed these symptoms suddenly after waking up from sleep. Her birth history was uneventful, and she achieved developmental milestones on time. The patient occasionally experienced fatigue during play, with ptosis improving after sleep. Family history, allergies, and past medical/surgical issues were unremarkable. She reported intermittent body aches and tiredness, denying shortness of breath or sleep apnea.

On examination, the patient appeared pale and lethargic, with evident ptosis and exo-deviation in the right eye. Upper limb reflexes were normal, while lower limb reflexes were weak. Gait was normal, and no facial palsy was observed. 

**Figure 1:**
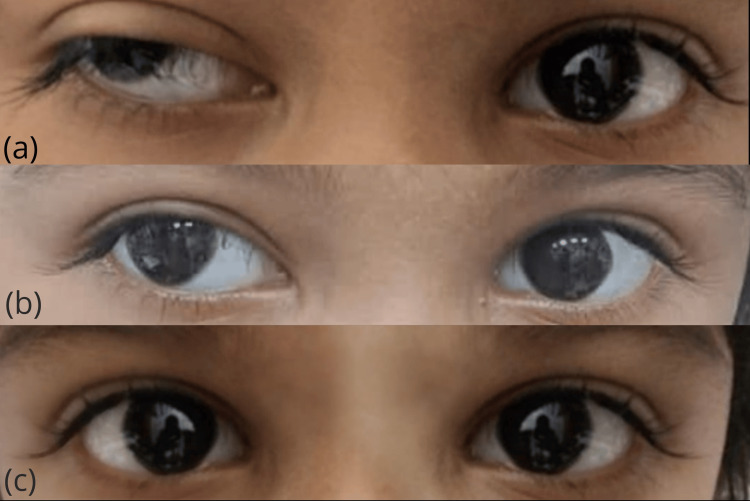
Photograph of the patient showing unilateral ptosis and exo-deviation of the right eye (a) at presentation, (b) one month after the initiation of the treatment, and (c) three months after the initiation of treatment

Ocular examination confirmed ptosis and outward deviation of the right eyeball (Figure 1a). The patient had meibomian gland dysfunction in both eyes on slit lamp bimicroscopy. However, the pupils, conjunctiva, cornea, anterior segment, lens, and posterior pole are all normal. Ptosis examination (Table 1) and orthoptic assessment (Table 2) revealed significant findings.

**Table 1:** Ptosis assessment in a four-year-old child with unilateral ptosis and exotropia NAD - no abnormality detected; mm - millimeters

Ptosis examination	Right eye	Left eye
Palpebral fissure height	4 mm	10 mm
Marginal reflex distance	-2 mm	4 mm
Levator function	4 mm with frontalis overaction	12 mm
Bell's phenomenon	Good	Good
Cogan twitch	Positive	NAD
Ice pack test	Positive (improved ptosis)	NAD
Lagophthalmos	NIL	NIL
Marcus Gun Jaw	NIL	NIL

**Table 2:** Orthoptic assessment of a four-year-old child with unilateral ptosis and exotropia XT - exotropia

Orthoptic assessment	Right eye	Left eye
Hirshberg test	30° exotropia	Straight
Cover test	XT + cyclotorsion	Straight + dominance
Krimsky test	60 prism dioptres	
Extraocular movements	Limited adduction, levodepression, levoelevation	Full
Abnormal head posture	Head tilt towards right	
Stereopsis	Nil (right eye suppression)	

The patient underwent a comprehensive evaluation for myasthenia gravis. While most baseline tests were unremarkable microcytic hypochromic anemia was detected. Further investigations like thyroid function tests, creatinine phosphokinase, renal function tests, liver function tests, and a chest X-ray for thymus enlargement all yielded normal results. Additionally, a computed tomography (CT) scan of the brain revealed no abnormalities.

The patient's blood tests revealed some concerning findings alongside reassuring ones. The hemoglobin (7.2 g/dL) and hematocrit (22.5%) were significantly lower than the normal range, indicative of anemia. This was further supported by the low mean corpuscular hemoglobin (17.5 pg) and mean corpuscular volume (54.9 fL) values, suggesting microcytic hypochromic anemia. Additionally, the alkaline phosphatase (356 U/L) was only mildly elevated. The test for acetylcholine receptor antibodies was borderline (0.45 nmol/L), warranting further testing.

The patient was booked for neostigmine testing to be performed in the operation room. The results of the neostigmine test were consistent with a diagnosis of MG. The palpebral fissure height (PFH) had improved by 7 mm; it was 3 mm before and 10 mm after the test. The electromyography (EMG) was performed which showed decremental response to repeated stimuli. 

The patient was diagnosed with OMG based on her symptoms, positive signs, and extensive diagnostic workup. She was prescribed oral pyridostigmine 60 mg at a dose of 6.9 mg/kg/day daily, divided into six doses by the pediatrician. Additionally, oral steroids were prescribed at a dosage of 20 mg per day, divided into two doses. Treatment for anemia was also initiated with anti-helminthics and iron supplements. The patient was followed up after one month and three months. After one month of treatment, the patient showed significant improvement in ptosis (Figure 1b). However, the exotropia did not fully improve, and she was advised to continue the same treatment to observe further results. After three months, the patient had a remarkable recovery and showed completely straight eyes (Figure 1c). The steroids were tapered, and pyridostigmine was continued with the same dosage. Hemoglobin after three months was improved to 10.8 g/dL. Alkaline phosphatase was also within normal range on follow-up.

## Discussion

MG is an autoantibody-mediated disorder compromising N-cholinergic transmission at the neuromuscular junction and manifests as fluctuating muscle weakness [4]. OMG, a subtype of MG, primarily affects the eye muscles. Painless ptosis, diplopia, and extrinsic ophthalmoparesis should raise suspicion of OMG. While OMG often presents with asymmetric muscle weakness and symptom fluctuations, it can resemble isolated cranial nerve paresis, internuclear ophthalmoplegia, or conjugate gaze palsy [5]. MG features fluctuating muscle weakness, often worsening with muscle use. It is known for affecting eyes, respiratory muscles, bulbar structures, or limbs. Initial complaints, like drooping eyelids and double vision, occur in over 50% of patients, earning MG the nickname "the great imitator" for its diverse presentation mimicking neurological disorders [6].

Khan et al. (2016) also reported a case with an unusual presentation of OMG, which was remarkably similar to our case. The reported case showed unilateral external ophthalmoplegia without accompanying symptoms like generalized weakness. Treatment with pyridostigmine and prednisolone led to significant improvement within a week, highlighting the efficacy of standard therapy in atypical MG cases [7]. Our case showed unilateral ptosis and exo-deviation OD and showed dramatic improvement with standard therapy. 

Colavito et al. reported that about two-thirds of MG cases initially present with ptosis. MG may manifest without ptosis and affect non-striated muscles, presenting as non-strabismic vergence anomaly or comitant non-variable strabismic deviation. OMG diagnosis relies on patient history, clinical findings, and diagnostic procedures [8]. According to Afifi et al., ptosis is the most common initial sign in MG patients. They found that 33% initially had unilateral ptosis, and in 11%, it remained unilateral throughout the disease course [9]. Evoli et al. found in their study that ptosis in MG is generally symmetrical, with about 35% of patients experiencing conjugated gaze paresis. Treatment with prednisone showed a positive response [10]. In our case, the patient developed acquired unilateral ptosis and exo-deviation that persisted and was the initial presenting complaint of the patient, which responded well to steroid treatment. Sri-udomkajorn et al. found that MG can present as unilateral ptosis or facial drooping without typical fluctuating muscle weakness. Patients with OMG had an earlier onset compared to those with generalized MG (GMG), as highlighted in the study. Their study also showed that 99% of patients had ptosis, 67% of which had bilateral and 33% had unilateral ptosis [11]. Our patient was four years old (early age), had no systemic features of MG (isolated OMG), presented as unilateral ptosis and exo-deviation, and showed no fluctuation of symptoms with progression of the day; however, the ptosis would improve upon waking up. 

MG, however, rarely can present with ptosis as an isolated finding. A retrospective review by Donaldson et al. on undifferentiated ptosis in a neuro-ophthalmology practice found no MG in patients with isolated unilateral ptosis and normal examinations. This suggests investigating isolated ptosis for MG, which has a low yield [12]. The case we present is of a young child with acquired disease onset and no prior complaints; it is a rare MG presentation worth reporting.

The icepack test in our patient was positive, which gave us an initial clue to the diagnosis. Golnik et al. conducted an ice test on 20 MG patients and 20 without MG. Results showed that 80% of MG patients had a positive ice test, while none in the non-MG group did. Among four MG patients with complete ptosis, three had a negative ice test result [13]. This suggests that the ice test is a simple, short, specific, and relatively sensitive test for diagnosing myasthenic ptosis, but the sensitivity of the ice test decreases considerably in patients with complete ptosis.

The absence of anti-AChR antibodies in MG is notable, especially in cases with isolated eye involvement, steroid history, or thymectomy. Anti-AChR levels are influenced by factors like thymoma, age at onset, and steroid therapy. Thymomas are linked to elevated anti-AChR and anti-SM antibodies, revealing complex dynamics in MG [14]. Our patient's anti-AChR levels within the intermediate range may signify eye-limited disease, offering a nuanced perspective on MG pathology. 

Poudel et al. reported a similar case to ours involving a seven-year-old girl with seropositive juvenile GMG. Both patients presented with unilateral blepharoptosis, positive icepack and neostigmine challenge tests, and AChR antibody positivity. Thymic abnormalities were absent, and pyridostigmine and oral prednisolone were used. Their case received high-dose pyridostigmine and oral prednisolone, which were ineffective. They achieved success with monthly pulse intravenous methylprednisolone, daily oral prednisolone, and continued pyridostigmine without significant side effects [15]. Our patient, however, responded to the low levels of steroids and maximum dose of pyridostigmine. Both cases demonstrate the importance of individualizing treatment strategies based on patient response and also highlight the potential of pulse intravenous methylprednisolone for refractory juvenile MG cases when standard therapies fall short.

OMG, diagnosed earlier in children, can benefit from early prednisolone use. Studies suggest starting with a high daily dose of 50-60mg of prednisone and gradually tapering to maintenance doses (around 10mg or less) effectively resolves ptosis and diplopia. This approach achieves long-term remission in approximately 70% of cases for at least two years [16]. Our patient was started on low-dose steroids and responded quite well, which points out that children can benefit from low-dose steroids as well.

The limitation of our study is that anti-MuSk antibodies couldn't be performed due to financial constraints. More cases with similar findings need reporting to help physicians diagnose MG with diverse presenting complaints and improve the morbidity and mortality of patients with MG. 

This case report highlights an uncommon presentation of myasthenia gravis in a four-year-old, featuring persistent unilateral ptosis and exo-deviation. Diagnostic challenges were met with thorough testing, and successful treatment with pyridostigmine and corticosteroids underscored the importance of individualized approaches. The case emphasizes early recognition for improved outcomes in MG.

## Conclusions

OMG is the initial presentation of GMG in about half of the patients with MG. MG has an extensively variable presentation, therefore, high degree of suspicion and lower threshold for diagnosis of MG should be kept particularly in pediatric population to prevent missing of the cases and minimize morbidity and mortality in patients with myasthenia gravis. Early detection of MG can lead to better quality of life and increase life expectancy in patients with autoimmune diseases like MG.
